# Brown Tumor of the Dorsal Spine With Hemorrhage Causing Acute Neurological Deterioration: A Rare Presentation of Secondary Hyperparathyroidism

**DOI:** 10.7759/cureus.63645

**Published:** 2024-07-02

**Authors:** Siddharth Srinivasan, Bharat Raju, Rajesh Nair, Ajay Hegde, Sarah Johnson, Girish Menon

**Affiliations:** 1 Neurosurgery, Kasturba Medical College, Manipal, Manipal, IND; 2 Neurosurgery, Manipal Hospitals, Bangalore, IND; 3 Neurosurgery, Mayo Clinic, Rochester, USA; 4 Neurosurgery, Kasturba Medical College, Manipal, Udupi, IND

**Keywords:** chronic kidney disease, secondary hyperparathyroidism, hemorrhage, spine, brown tumor

## Abstract

Brown tumor due to secondary hyperparathyroidism in chronic kidney disease is a well-established entity. Brown tumor of the spine with hemorrhage causing acute neurological deficit is a rare entity.

A 35-year-old gentleman, with chronic kidney disease (CKD) on dialysis, presented with acute paraplegia and loss of lower limb sensation and bowel and bladder control. Imaging revealed a T8 vertebral body expansile lytic lesion with collapse, exaggerated kyphosis, and cord compression. He underwent an emergency decompressive laminectomy and transpedicular corpectomy of T8, with posterior stabilization. Histopathology revealed lobular clusters of osteoclast-like multinucleated giant cells with background of which was possibly the reason for acute neurological deterioration in this case.

Brown tumors of the spine can mimic lytic lesions of the spine like myeloma and metastasis. Suspicion must be raised given in the setting of CKD and hyperparathyroidism. They can present with hemorrhage and acute neurological deficit, which warrants urgent surgical intervention for optimal outcomes.

## Introduction

Brown tumor is a benign bone lesion resulting from primary or secondary hyperparathyroidism [1]. It arises due to osteoclastic overactivity and trabecular fibrosis induced by a chronic increase in serum parathormone. Although rare in the spine, brown tumors can mimic other conditions such as myeloma, leukemia, and metastasis. However, in patients with chronic kidney disease (CKD) and secondary hyperparathyroidism, suspicion of a brown tumor should be heightened [2]. Secondary hyperparathyroidism occurs due to a compensatory increase in serum parathormone levels following hypocalcemia, commonly caused by CKD, vitamin D deficiency, and inadequate intake. The first reported case of a spinal brown tumor was by Shaw and Davies in 1968 [3]. Clinical manifestations can vary from dull aching pain and myelopathy to flaccid paralysis, loss of sensation, and bowel and bladder incontinence. These tumors typically have a predilection for the facial bones and mandible. In this report, we discuss a case of brown tumor of the thoracic spine, its presentation, and its management.

## Case presentation

**Figure 1:**
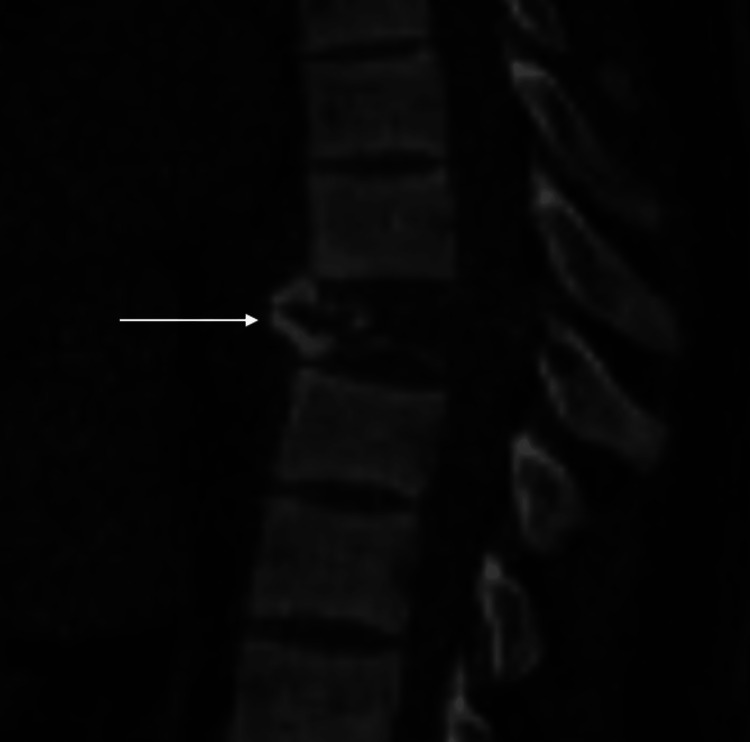
Computed tomography (CT) of the spine sagittal, T8 vertebral body expansile lytic lesion with collapse and impending kyphosis, as indicated by the arrow

**Figure 2:**
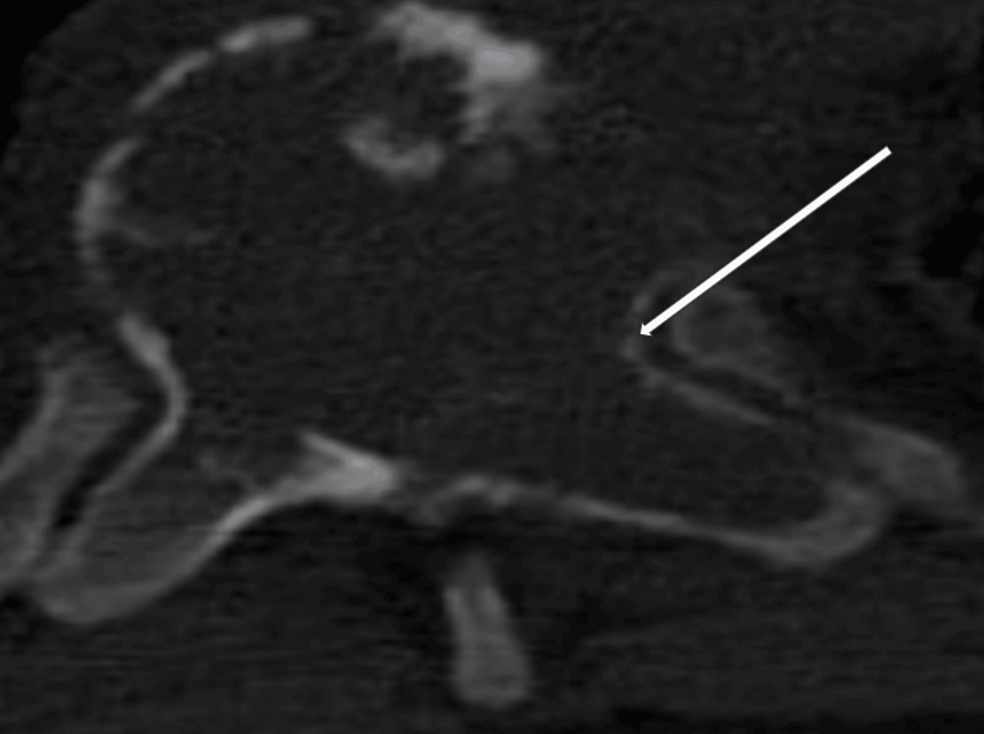
Computed tomography (CT) of the spine axial view, T8 lytic lesion involving the vertebral body, extending into the pedicle (arrow) and lamina

A 35-year-old gentleman, with a known case of chronic kidney disease (stage 5d) on regular dialysis, presented with a history of acute paraplegia, loss of sensation below his umbilicus, and bowel and bladder incontinence for six hours. He was categorized as American Spinal Injury Association Score - A (ASIA). Computed tomography (CT) of the spine revealed an expansile lytic lesion in the T8 vertebral body with collapse, extending to involve the pedicles and laminae (Figures 1, 2).

**Figure 3:**
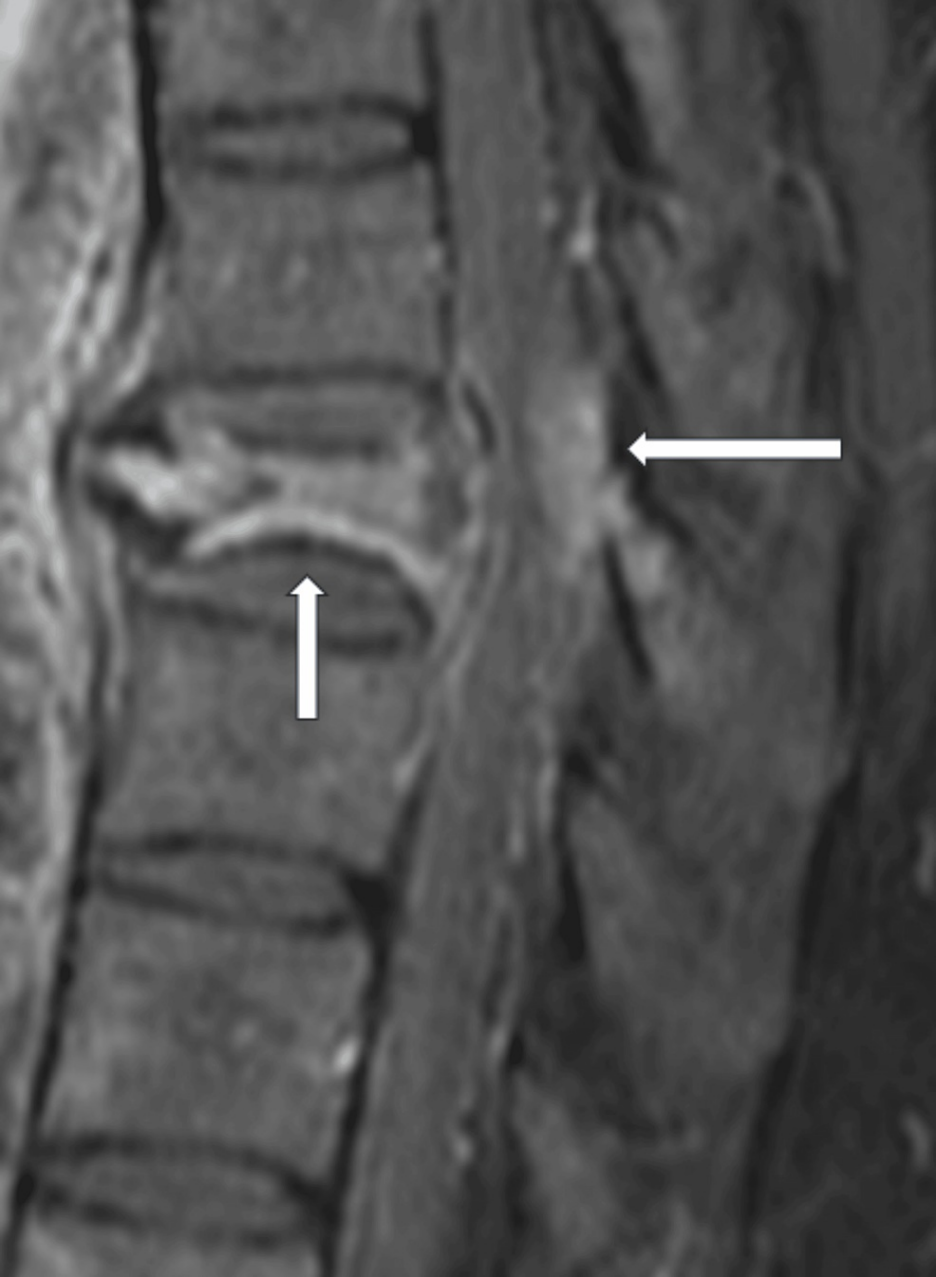
T1- Post-gadolinium contrast - magnetic resonance imaging (MRI), mid-sagittal view of the spine demonstrating a heterogenous contrast-enhancing lesion involving the body with collapse (vertical arrow) with significant spinal cord compression (horizontal arrow)

Magnetic resonance imaging (MRI) of the spine depicted a heterogeneous contrast-enhancing lesion with significant spinal cord compression and signal changes in the cord (Figure 3).

Considering the patient's clinical background of chronic kidney disease and hyperparathyroidism, a working diagnosis of a brown tumor of the spine with compressive dorsal myelopathy was established. However, the acute neurological deterioration without prior symptomatology was unusual for this diagnosis.

**Figure 4:**
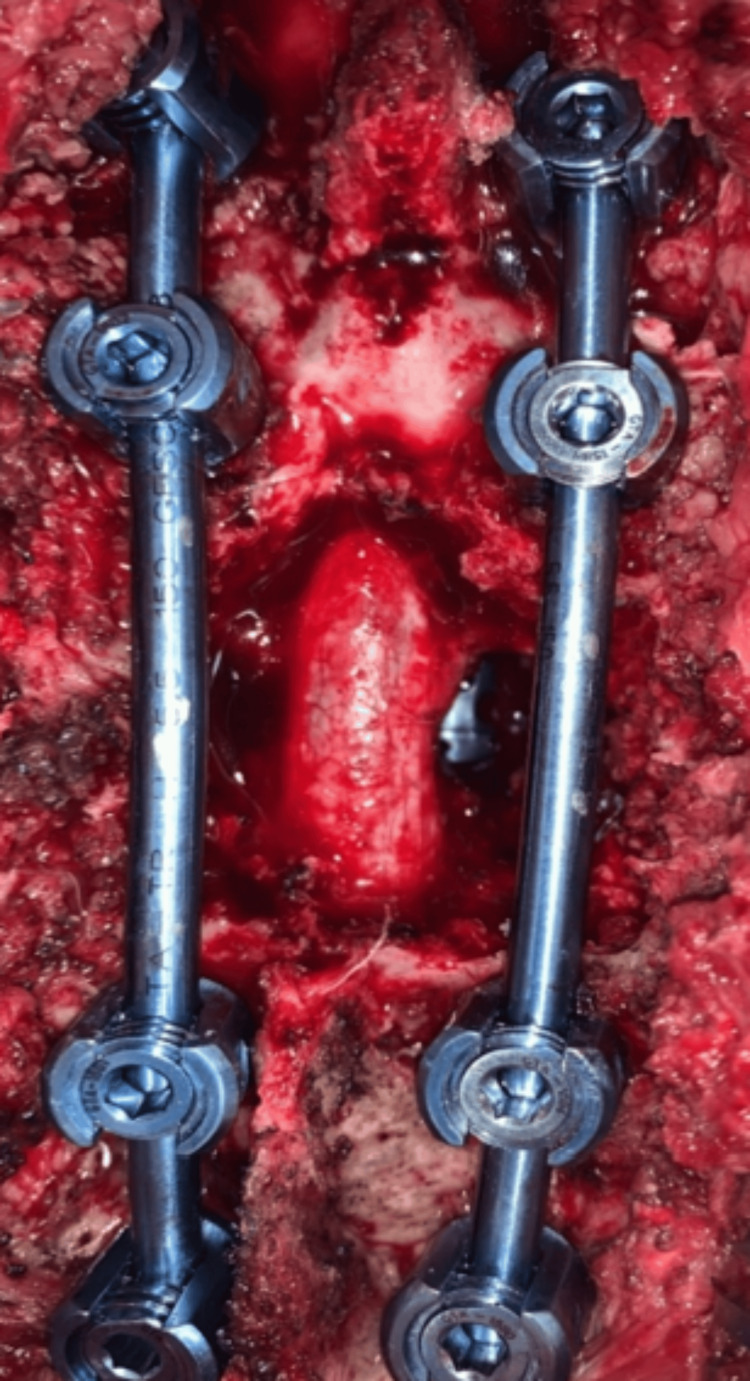
T6, T7, T9, T10 pedicle screw stabilization, cage visible in the depth; adequately decompressed spinal cord noted

His serum parathormone level was 1290 pg/ml (normal range: 20-70 pg/ml). Serum calcium was 6.9 mg/dl (normal range: 8-10 mg/dl), and serum phosphate was 5.5 mg/dl (normal range: 2.5-4.5 mg/dl). He underwent emergency dorsal spine decompression surgery, tumor decompression, and instrumented stabilization. A T8 transpedicular corpectomy with expandable spacer insertion was performed to prevent worsening kyphosis. T6, T7, T9, and T10 pedicle screw stabilization, along with posterior T7, T8, and T9 laminectomy, was done to decompress the spinal cord (Figure 4).

Intraoperatively, there was evidence of hemorrhage within the tumor, which we assume to be the plausible cause of the acute neurological deterioration in this case. The spinal cord pulsated well after decompression. The bone around the implants was decorticated with a diamond burr and bone graft harvested from the excised spinous process and laminectomy was morcellated and placed around the screw-rod construct to facilitate fusion.

**Figure 5:**
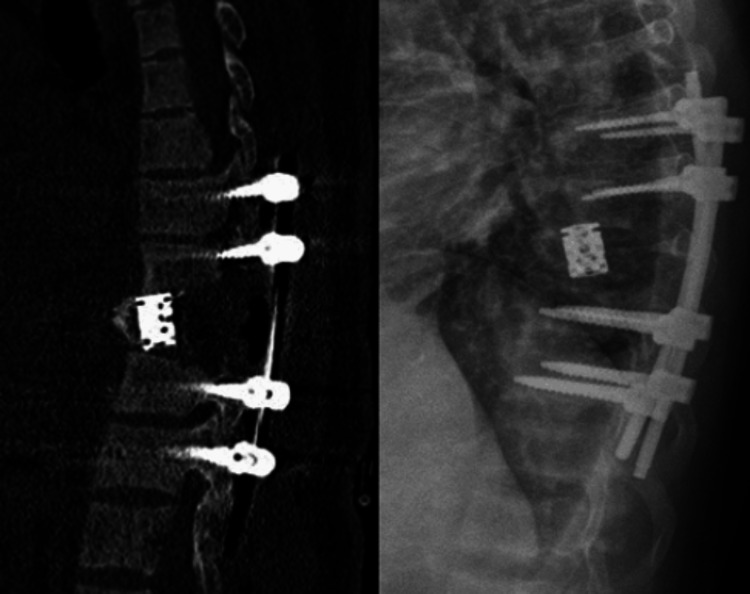
Postoperative CT and X-ray of the spine depicted adequate kyphosis correction and appropriate screw-rod construct positioning

Postoperative CT and X-ray of the spine showed adequate kyphosis correction and proper positioning of the screw-rod construct (Figure 5).

**Figure 6:**
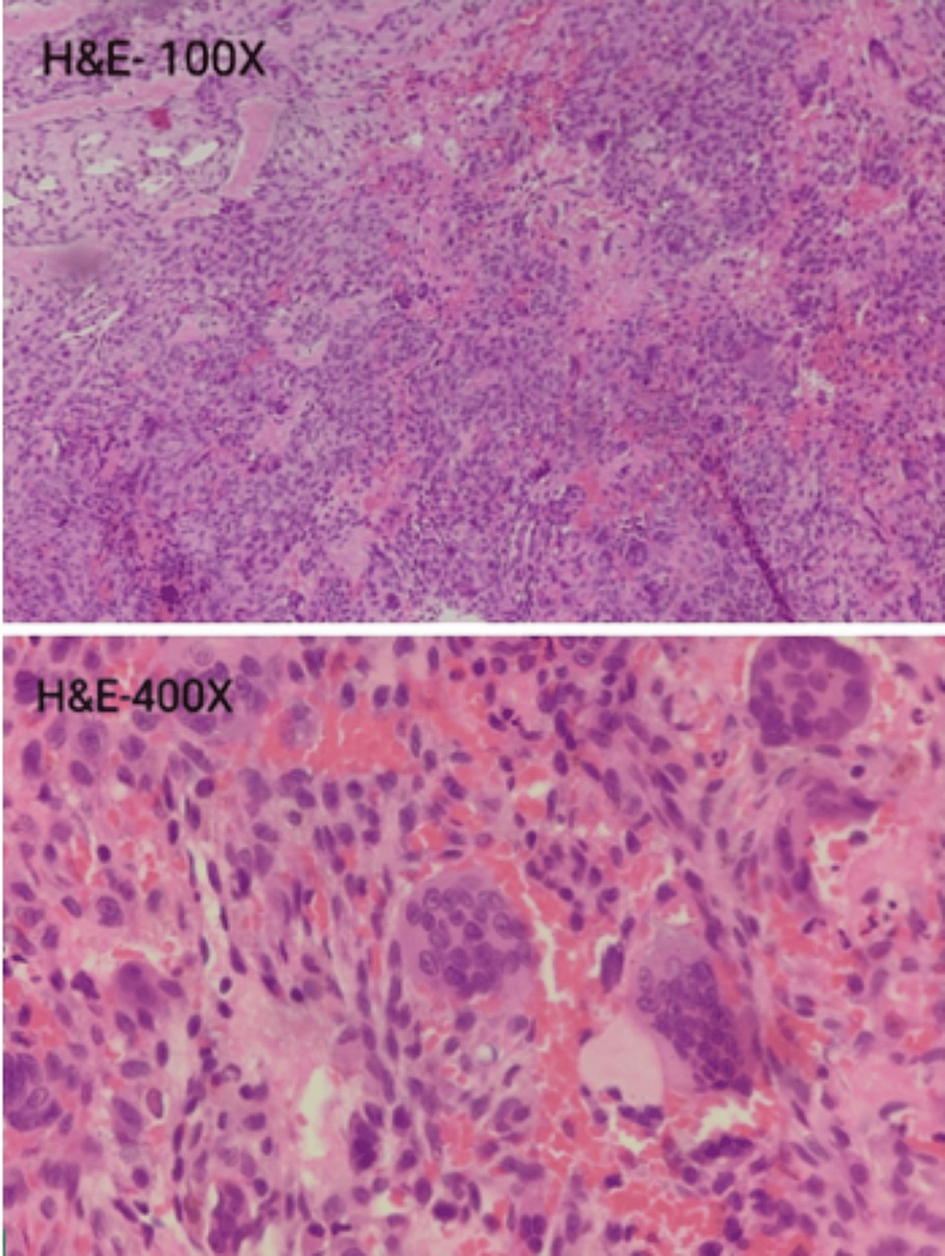
Histopathology revealed clusters of osteoclast-like multinucleated giant cells, hemosiderin-laden macrophages, and hemorrhages

Histopathology revealed clusters of osteoclast-like multinucleated giant cells, hemosiderin-laden macrophages, and hemorrhage (Figure 6).

For adjunct treatment, the patient was started on calcium supplements and oral cinacalcet. Early postoperative recovery included the return of crude touch sensations in his lower limbs (ASIA-B). However, his motor weakness and bowel/bladder incontinence persisted, and he is currently undergoing rehabilitation and physiotherapy. A postoperative technetium-99 sestamibi scan did not show parathyroid adenoma, confirming the diagnosis of secondary hyperparathyroidism due to CKD.

## Discussion

Brown tumor derives its name from the hemosiderin that gives the stroma a brownish hue [4]. It can be a presenting feature of primary, secondary, or tertiary hyperparathyroidism [1]. Brown tumors in general are more common in primary hyperparathyroidism, but brown tumors of the spine are more common in secondary hyperparathyroidism [5]. Brown tumor of the spine is a rare entity and to date from 1977 we found only 53 cases of spinal brown tumor in patients with primary or secondary hyperparathyroidism.

Bone disease in chronic kidney disease patients was classified into osteitis fibrosa (high turnover bone), adynamic bone disease (low bone turnover), and osteomalacia (inadequate mineralization) [6]. Brown tumors belong to the osteitis fibrosa group [7]. Most of them are present in the third or fourth decade of life [4]. They usually manifest as back pain, radiculopathy, and myeloradiculopathy with or without sphincter dysfunction [8]. Serum parathormone is almost always elevated and in patients with brown tumors, it is anticipated to be more than 10 times its normal value, as in our patient [9]. The clinical presentation and imaging features of a brown tumor can masquerade as myeloma, blood dyscrasias, spine metastasis, giant cell tumors, and aneurysmal bone cysts [10-12]. The diagnosis mustn't be missed since it is a completely treatable benign problem with favorable outcomes [13]. In primary hyperparathyroidism, a biopsy of the brown tumor is required since that may be the first manifestation of the disease. However, in secondary hyperparathyroidism, a biopsy may not be necessitated since the patient usually has other clinical manifestations that can direct to the possibility of a brown tumor.

Brown tumor has to be primarily managed medically for secondary hyperparathyroidism with calcimimetics, phosphate binders, low phosphate diet, and vitamin D analogs. As per the Kidney Disease Improving Global Outcomes (KDIGO) 2017 update, calcimimetic cinacalcet has unmitigated significance in treatment for optimizing parathormone levels. If patients do not improve on medical management alone then a parathyroidectomy should be considered, as it has definitively proven benefits in reducing PTH values, remineralization of bones, and resolution of brown tumors [14]. Spine decompression surgery and stabilization are warranted if the patient has progressively worsening neurological symptoms and bony instability on imaging as done in our case.

Hypervascular spinal tumors, such as atypical hemangioma, aneurysmal bone cysts, and metastasis from renal cell cancer and thyroid carcinoma, are common causes of epidural spinal hemorrhage [15]. Brown tumor presenting with a hemorrhagic presentation is a sporadic phenomenon. More importantly, we would like to emphasize the need for rigorous follow-up imaging, especially for patients who have been diagnosed with spinal brown tumor. This case report's overarching aim is to enlighten that the disease progression and treatment response must be vigilantly monitored. If the spinal brown tumor is progressing on imaging, depending on the degree of bony involvement, neurological deficits must be anticipated and timely preventive surgical measures must be advocated.

## Conclusions

Brown tumor of the spine is a rare disease, now being increasingly described in patients who are on hemodialysis. Hemorrhage in a brown tumor of the spine has not been described before and that could be the cause of acute neurological deterioration as in this case. Early diagnosis and intervention in the form of decompression with instrumented fusion techniques may be beneficial. Secondary hyperparathyroidism needs to be addressed to prevent the progression of the disease. Vigilant follow-up imaging is a must if a spinal brown tumor has been diagnosed, to facilitate timely intervention if progression is noted.
